# Focusing a viral risk ranking tool on prediction

**DOI:** 10.1073/pnas.2419337122

**Published:** 2025-04-17

**Authors:** Katherine Budeski, Marc Lipsitch

**Affiliations:** ^a^Department of Epidemiology, Harvard T.H. Chan School of Public Health, Boston, MA 02115; ^b^Center for Communicable Disease Dynamics, Harvard T.H. Chan School of Public Health, Boston, MA 02115

**Keywords:** spillover, zoonoses, zoonotic risk

## Abstract

Preparing to rapidly respond to emerging infectious diseases is critical. *SpillOver: Viral Risk Ranking* is an open-source tool developed to assess the risk of novel wildlife-origin viruses spilling over from animals to humans and spreading in human populations. Several risk factors used by the tool depend on evidence of previous zoonotic spillover itself or sustained transmission in humans. Therefore, we reanalyzed the *Ranking Comparison* after removing eight of the 31 risk factors that require postspillover knowledge and compared the adjusted risk rankings to the originals. The area under the receiver operating characteristic curve deteriorated from 0.94 for the original risk scores to 0.73 for the adjusted ones for predicting the classification as a human virus. We also compared the mean and SD of the risk scores for the human and non-human viruses at the risk factor level. Most excluded spillover-dependent risk factors had dissimilar means between the human and non-human virus classifications, but nonspillover-dependent risk factors frequently showed similar means between the two classifications. The original formulation of the tool depended on the inclusion of spillover-dependent risk factors to quantitatively assess the risk of zoonotic spillover for a novel virus. Future iterations of the tool should omit such risk factors and consider other nonspillover-dependent risk factors to ensure that the tool is fit for risk prediction of novel viruses.

The *SpillOver: Viral Risk Ranking* tool (Spillover tool) presents a publicly available, interactive platform “for policy makers, scientists and the general public to assess the likelihood that a wildlife virus will spillover and spread in humans” (1). Supported by PREDICT, a former project of the US Agency for International Development Emerging Pandemic Threats program (2), and the Global Virome Project (3), the Spillover tool has ranked over 800 wildlife-origin viruses (1). Published results of the original ranking exercise show that the top 12 viruses identified as having the highest risk of zoonotic spillover had indeed spilled over at the time of publication, a result described as “[v]alidating the risk assessment” (1). Inputs to the Spillover tool can evolve over time. As of August 13, 2024, the top 10 viruses ranked by the tool are all known to infect humans: SARS-CoV-2, Lassa, Ebola, Seoul, Nipah, Hepatitis E, Marburg, Simian Immunodeficiency, Rabies, and Lymphocytic Choriomeningitis viruses. However, several of the risk factors used to quantify a virus’ risk of spillover require prior evidence of zoonotic spillover itself or widespread transmission in humans. Given the expressed aim of the tool to evaluate novel zoonotic viruses, we performed a reanalysis of the Spillover tool, taking the data as downloaded from https://spillover.global/ranking-comparison/ but eliminating from the scoring those risk factors whose scores depended on the fact that the virus of interest had spilled over or caused widespread transmission in humans (1).

## Results

Eight of the 31 original risk factors were identified as partially or fully dependent on evidence of spillover of a virus of interest. We calculated adjusted risk scores identically to the original paper (1), as sums of weighted risk scores for the risk factors evaluated for each virus in the Spillover database but omitted the risk scores for the eight spillover-dependent risk factors. The original risk scores match those presented in the original analysis (1). Throughout the analysis, we maintained the use of the assigned expert weights as described by Grange et al. (1). For further information regarding the selected spillover-dependent risk factors and an overview of the Spillover tool’s methodologies including a description of the risk scores and weights, see the Supplementary Information.

**Fig. 1.**
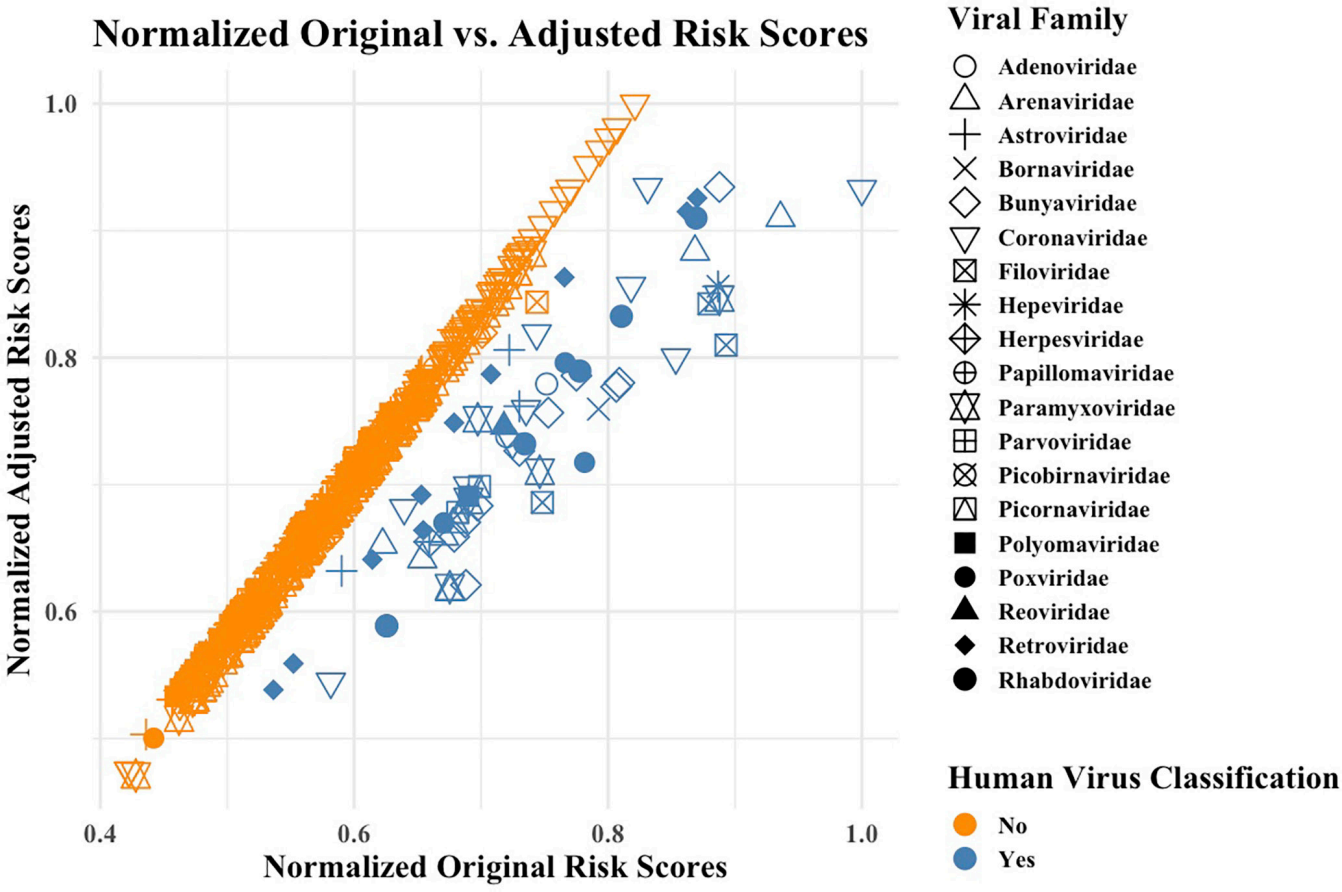
Normalized adjusted vs. normalized original risk scores. Viruses are coded by shape to represent their viral families and by color to indicate whether the virus was classified as a human virus or not as reported by the descriptive variable entitled “Human Virus?” within the Spillover tool.

Fig. 1 displays the normalized adjusted (*y*-axis) and normalized original (*x*-axis) risk scores for every virus listed within the *Spillover* tool’s *Ranking Comparison* before May 22, 2024. A strong near-diagonal relationship demonstrates that many viruses’ scores were affected modestly or not at all by the removal of the spillover-dependent risk factors, while the points to the right of the diagonal represent viruses whose normalized scores went down substantially after the removal of spillover-dependent risk factors. Viruses classified as human viruses in the Spillover database were more affected by the removal of the spillover-dependent factors than those classified as non-human viruses.

The original ranks of the top 10 viruses using the adjusted risk scores ranged from 1 (SARS-CoV-2) to 29 (Coronavirus PREDICT CoV-24), indicating that the top 10 viruses by the adjusted risk scores had also scored very high on their original risk scores.

Using the normalized adjusted and normalized original risk scores the discriminatory power quantified using the area under the receiver operating characteristic curve (AUROC) comparing viruses classified as human and non-human viruses by the binary variable entitled “Human Virus?” within the Spillover tool was calculated. The normalized original risk scores had an AUROC of 0.94 versus 0.73 for the normalized adjusted ones. Therefore, the removal of spillover-dependent risk factors makes the adjusted risk scores considerably less strongly associated with observation of human infections than the original risk scores.

**Fig. 2.**
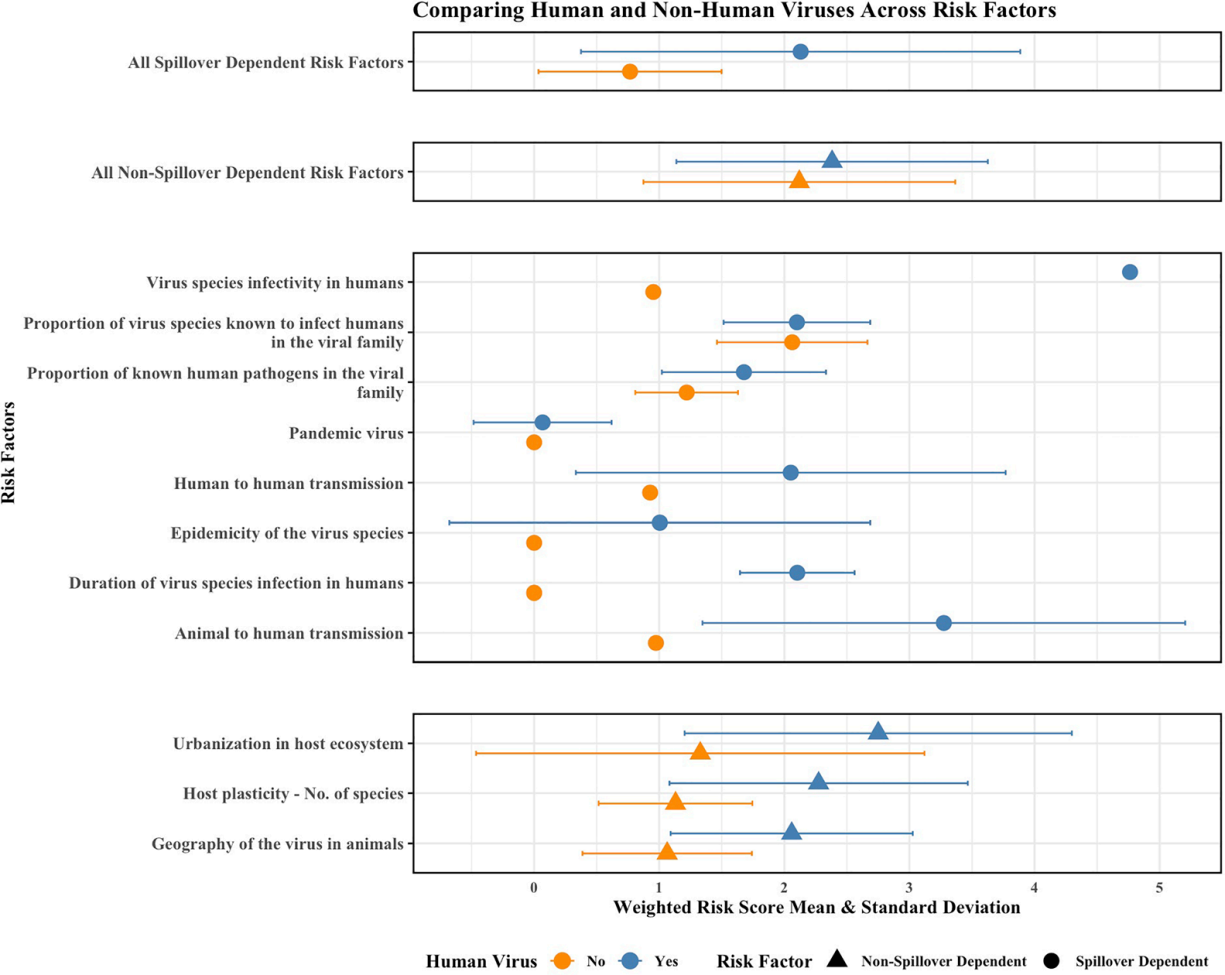
Weighted mean risk scores and their SD for human (blue) and non-human (orange) viruses for the aggregated spillover- and nonspillover-dependent risk factors as well as the individual eight spillover-dependent risk factors and the three nonspillover dependent risk factors with the most dissimilar means between the human and non-human viruses as classified by the Spillover tool. Circles indicate spillover-dependent risk factors and triangles indicate nonspillover-dependent risk factors.

Fig. 2 shows the mean risk scores and SD for human and non-human viruses by individual spillover-dependent risk factors and highlights how these factors contribute to the Spillover tool’s ability to “predict” that a novel virus will spillover and transmit among humans. The top three factors retained in the adjusted risk scores that were higher among the human viruses were: “Urbanization in the host ecosystem,” “Host plasticity - No. of species,” and “Geography of the virus in animals” as written in the Spillover tool (1).

## Discussion

As a virus spreads, we may learn more about its pathogenicity and transmissibility. In their 2021 paper, published the year after SARS-CoV-2 started a global pandemic, Grange et al. noted that SARS-CoV-2 was originally ranked in the *Ranking Comparison* second to Lassa virus (1). The authors credit the lower ranking to the lack of data available for SARS-CoV-2. Notably, the version of the tool now available online ranks SARS-CoV-2 as the virus with the highest risk of zoonotic spillover, providing further evidence of how the addition of postspillover data affects the original scoring system to favor viruses with a known spillover event.

The declared goal of the Spillover tool is to “systematically evaluate novel wildlife-origin viruses in terms of their zoonotic spillover and spread potential” (1). A tool for that purpose should include only risk factors that are available and relevant for novel wildlife-origin viruses and not factors that are available only after spillover and/or widespread transmission has been observed. Validating the tool using spillover-dependent input data and finding that the tool’s output matches observed spillovers risks circularity.

Our analysis shows that the top 10 threats identified by the tool change when postspillover criteria are omitted, replacing 8 of the original top 10 with 7 coronaviruses and 1 hantavirus that are not yet known to have spilled over into humans, replacing one with a coronavirus (229E) that has occurred in humans (4), and preserving SARS-CoV-2, which moved from the first to the seventh rank (Fig. 1). While the adjusted scoring system maintains the predictive ability for viruses that are known to have spilled over, it is substantially less predictive than the scoring system that incorporates knowledge of spillover among the risk factors.

Furthermore, expert opinion ranked as most important for spillover risk (highest *Risk Factor Influence*) some spillover-dependent factors (including the top three highest-ranked risk factors) in the final score (1). By calculating the mean and SD for all spillover- and nonspillover-dependent risk factors among human and non-human viruses, we were also able to observe some risk factors that were not obviously spillover-dependent yet did contribute to the distinction between the human and non-human virus groups. The three nonspillover-dependent risk factors with the most dissimilar means between the human and non-human viruses (shown in Fig. 2) highlight that some nonspillover-dependent factors may be especially informative for assessing the risk of spillover.

Tools to rapidly quantify the risk posed by a novel virus will only become increasingly relevant as the threat posed by infectious diseases is exacerbated by climate change, globalization, and political, social, and economic instability (5–7). However, it is not obvious how to validate a tool designed to assess the threat posed by infrequent future events. An ideal evaluation might assess factors over time and calculate the distribution of scores at various time points in the past when spillover events happened, asking whether those that spilled over in the past were rated highly likely to spill over with data available at that time—though reconstructing previous states of knowledge might be challenging in practice. Plowright et al. highlight that multiple factors must align to permit spillover, suggesting that an additive model of individual factors may be less useful than one with functional flexibility to account for interactions of multiple factors (8). Similar assessments could be conducted to evaluate the risk of a novel virus spreading in human populations. However, many additional factors should be considered (beyond those identified in the Spillover tool) to assess the risk that a novel virus will spread in human populations including the availability of medical countermeasures, baseline population level immunity, and a quantification of the level of pandemic preparedness in the countries experiencing the outbreak among other factors. We encourage further methodological refinement to better evaluate such tools, rigorous adherence to using criteria known prior to spillover, and further exploration of the predictive power of the risk factors that are not spillover-dependent but are nonetheless relevant for viruses that have spilled over.

## Materials and Methods

The published *Virus Risk Ranking Assessment* included 31 factors in their final *Spillover Risk Score* (1). All risk factors that would have a higher score if the virus in question had a known spillover event or shown widespread transmission within humans were identified. Eight risk factors were flagged to have met this spillover-dependent criterion (SI Appendix). Once these risk factors were identified the adjusted risk scores and risk rankings were recalculated without them. The adjusted risk score included all 31 risk factors except the eight spillover-dependent factors. The original risk scores match those of the Spillover tool and include all 31 risk factors. The expert weights were maintained for each factor as determined by the *Risk Factor Influence* described by Grange et al. (1). Additional details of the data analysis can be found in Supplementary Information.

## Supplementary Material

Appendix 01 (PDF)

## Data Availability

The data used for this analysis is publicly available and can be downloaded at https://spillover.global/ranking-comparison/. All associated code used for this analysis can be found at https://github.com/c2-d2/Focusing-a-Viral-Risk-Ranking-Tool-on-Prediction-2025. All figures can be reproduced using the instructions and code provided in the repository. Previously published data were used for this work (1).
