# Supplementary material for: Focusing a viral risk ranking tool on prediction
Source: Proc Natl Acad Sci U S A. 2025 Apr 17;122(16):e2419337122. doi: 10.1073/pnas.2419337122 (PMC12036980; doi:10.1073/pnas.2419337122)
Supplement: Supplementary file 1 — Appendix 01 (PDF) [file pnas.2419337122.sapp.pdf]

Supplementary Information

Description of the Spillover Tool's Risk Ranking Methods

The *SpillOver: Viral Risk Ranking* tool (Spillover tool) was developed as a "comparative risk assessment" for "viruses of uncharacterized zoonotic spillover potential alongside those already known to be zoonotic" (1). The risk assessment was developed with a literature review and expert opinion to identify and weigh those risk factors that contribute to the risk of a virus to spillover from animals to humans, as described by Grange et al. (1). The identified risk factors were previously evaluated for each virus in the Spillover tool's database producing a *Spillover Risk Score*, referred to as the risk score, for each virus (1). There are 31 risk factors across three categories related to the host, virus, and environment that contribute to the risk score for each virus. The risk factors, as written in the Spillover tool, are:

- *Host plasticity - No. of species,*
- *Host plasticity - No. of orders,*
- *Geography of the host(s),*
- *Number of primary high-risk disease transmission interfaces where the virus has been detected,*
- *Genetic relatedness between the host species and humans,*
- *Land use in host ecosystem,*
- *Livestock density in host ecosystem,*
- *Human population density in host ecosystem,*
- *Deforestation in host ecosystem,*
- *Urbanization in host ecosystem,*
- *Agricultural system change in host ecosystem,*
- *Genome classification of the virus,*
- *Envelope status of the virus,*
- *Viral genome segmentation,*
- *Virus species infectivity in humans,*
- *Virus species infectivity in terrestrial mammals (excluding humans),*
- *Proportion of virus species known to infect humans in the viral family,*
- *Proportion of virus species known to infect terrestrial mammals (excluding humans) in the viral family,*
- *Proportion of viruses within a viral family that are known to infect more than 1 host species,*
- *Epidemicity of the virus species,*
- *Geography of the virus in animals,*
- *Proportion of known human pathogens in the viral family,*
- *Transmission mode of the virus,*
- *Animal to human transmission,*
- *Human to human transmission,*
- *Duration of virus species infection in humans,*
- *Pandemic virus,*
- *Frequency of interaction between domestic animals and humans in the host ecosystem,*

- *Intimacy of interaction between domestic animals and humans in the host ecosystem*,
- *Frequency of interaction between wild animals and humans in the host ecosystem*, and
- *Intimacy of interaction between wild animals and humans in the host ecosystem* (1).

The *Risk Level Score*, or possible score assigned for each individual risk factor, ranges on a scale between 0 and 5 (1).

Next, the weight of each risk factor was determined by what Grange et al. term *Risk Factor Influence* (1). According to Grange et al., 65 experts contributed to determining the weight of each risk factor. Experts were asked to both assign their perceived level of *Spillover Risk*, meaning how relevant each expert thought the risk factor was for spillover and to self-report their *Level of Expertise* on each risk factor they assessed. The *Risk Factor Influence* was then the weighted average of the expert evaluation of the *Spillover Risk* for that risk factor, weighted by the experts' self-declared *Level of Expertise* on a scale of 1 to 16 (1). It should be noted that the experts were originally presented with 50 risk factors to evaluate as contributing to the risk that a wildlife-origin virus will spill over from animals to humans. Seven of those 50 risk factors were "assessed by experts as worthy of consideration but were eliminated due to the insufficiency of available data sources" (1). Finally, all other risk factors that scored less than a two on their *Risk Factor Influence*, demonstrating minimal importance to the risk of spillover, were excluded from the list of risk factors contributing to the risk score (1). Two risk factors were the exception to this cut-off: "Envelope status of the virus" and "Viral genome segmentation" (1). Furthermore, while the risk score is calculated using the 31 risk factors resulting from expert evaluation, the Spillover tool's database does provide information on up to 42 risk factors depending on the availability of data (1). The 31 risk factors used to calculate the risk score were the target of our evaluation.

This Analysis

First, we identified risk factors that would have a higher score if the virus in question has had a known spillover event or has shown widespread transmission within humans. Eight risk factors in the Spillover tool met this criterion including:

- *Animal to human transmission*,
- *Human to human transmission*,
- *Duration of infection in humans*,
- *Viral infectivity in humans*,
- *Epidemicity of the virus*,
- *Pandemic virus*,
- *Proportion of viruses known to infect humans in the viral family*, and
- *Proportion of known human pathogens in the viral family* (1).

These risk factors are referred to throughout our analysis as the "spillover-dependent risk factors," while the other 23 risk factors are referred to as "non-spillover-dependent risk factors."

Next, throughout our analysis, the weighted risk scores were used. The weighted risk scores were calculated, as described by Grange et al. by multiplying the *Risk Factor Influence* obtained from expert elicitation, which ranged from 0 to 3, by the *Risk Level Score* for each risk factor, which ranged between 0 and 5, and then dividing the product by 3 (1).

We calculated the adjusted risk scores by summing the weighted *Risk Level Scores* of the non-spillover-dependent factors for each virus in the Spillover tool's database, and both the original and adjusted risk scores were normalized relative to the highest risk score by each set of risk factors for comparability. The viruses with the top ten highest and lowest original and adjusted risk scores were identified (see Associated Code). The top 10 ranked viruses using the adjusted

risk scores were: Rousettus bat coronavirus HKU9 (73.9), Murine coronavirus (72.5), Chaerephon bat coronavirus/Kenya/KY22/2006 (72.0), Coronavirus PREDICT CoV-35 (71.2), Longquan Aa mouse coronavirus (70.3), Seoul virus (69.1), Coronavirus 229E (Bat strain) (69.1), Eidolon bat coronavirus/Kenya/KY24/2006 (69.0), Severe acute respiratory syndrome coronavirus 2 (69.0), and Coronavirus PREDICT CoV-24 (68.6).

The normalized original and normalized adjusted risk scores were plotted by the virus family and human virus characterization as classified in the Spillover tool's database (Fig. 1). Using the normalized adjusted and normalized original risk scores, the discriminatory power was quantified using the area under the receiver operating characteristic curve (AUROC) comparing viruses classified as human and non-human viruses in the Spillover tool's database using the pROC package (2). A human virus was defined as a virus with demonstrated capability to infect humans, as described by Grange et al. in their Supplementary Information (1). This category was chosen over the indicator for known human-to-human transmission as the Spillover tool aims to evaluate "novel" viruses that may or may not have caused known outbreaks in human populations despite the utilization of this information in the tool. Lastly, the mean weighted risk scores and their standard deviations for the aggregate of the risk factors were plotted by their spillover or non-spillover dependent classification as well as all eight individual spillover-dependent factors and the three individual non-spillover dependent risk factors with the most dissimilar means between the human and non-human viruses as classified by the Spillover tool's database (Fig. 2).

The original publicly available CSV was organized using a Jupyter Notebook (3) run in Google Colab. Pandas was the main library used for data manipulation (4). All further data visualizations and analyses were run using an R script (5) in R Studio Version 4.2.3 (6). The tidyverse and dplyr packages were used for data manipulation and visualization (7).

The data used for the analysis was downloaded as a CSV file on May 22, 2024, from https://spillover.global/ranking-comparison/. Therefore, the analysis only contains viruses submitted to the platform before this date.

OpenAI GPT 4o, ran through the Harvard AI Sandbox, assisted in the initial drafting of both the Python and R scripts used for this analysis (8). All draft code has been manually modified, expanded upon, and reviewed to meet the specific objectives of this analysis. All final code was tested to ensure the accurate reproducibility of the workflow associated with this analysis.

Supplementary References

1. Z. L. Grange, *et al.*, Ranking the risk of animal-to-human spillover for newly discovered viruses. *Proceedings of the National Academy of Sciences* **118** (2021).
2. X. Robin, *et al.*, pROC: an open-source package for R and S+ to analyze and compare ROC curves. *BMC Bioinformatics* **12** (2011).
3. B. E. Granger, F. Perez, JuPyter: Thinking and storytelling with code and data. *Computing in Science & Engineering* **23**, 7–14 (2021).
4. The pandas development team, pandas-dev/pandas: Pandas. (2024). Deposited 2024.
5. R: The R Project for Statistical Computing. Available at: https://www.r-project.org/.
6. PoSIt | the Open-Source Data Science Company. *Posit* (2025). Available at: http://www.rstudio.com/.
7. D. Vaughan, K. Müller, L. Henry, H. Wickham, R. François, A Grammar of Data Manipulation. (2023). Deposited 2023. https://github.com/tidyverse/dplyr
8. OpenAI GPT- 4o. *OpenAI.* (2024). https://sandbox.ai.huit.harvard.edu/
